# Reality in a sphere: A direct comparison of social attention in the laboratory and the real world

**DOI:** 10.3758/s13428-021-01724-0

**Published:** 2021-12-16

**Authors:** Jonas D. Großekathöfer, Christian Seis, Matthias Gamer

**Affiliations:** Department of Psychology, Julius Maximilian University of Würzburg, Würzburg, Germany

**Keywords:** Social attention, Spherical videos, Eye tracking, Ecological validity, Virtual reality

## Abstract

Humans often show reduced social attention in real situations, a finding rarely replicated in controlled laboratory studies. Virtual reality is supposed to allow for ecologically valid and at the same time highly controlled experiments. This study aimed to provide initial insights into the reliability and validity of using spherical videos viewed via a head-mounted display (HMD) to assess social attention. We chose five public places in the city of Würzburg and measured eye movements of 44 participants for 30 s at each location twice: Once in a real environment with mobile eye-tracking glasses and once in a virtual environment playing a spherical video of the location in an HMD with an integrated eye tracker. As hypothesized, participants demonstrated reduced social attention with less exploration of passengers in the real environment as compared to the virtual one. This is in line with earlier studies showing social avoidance in interactive situations. Furthermore, we only observed consistent gaze proportions on passengers across locations in virtual environments. These findings highlight that the potential for social interactions and an adherence to social norms are essential modulators of viewing behavior in social situations and cannot be easily simulated in laboratory contexts. However, spherical videos might be helpful for supplementing the range of methods in social cognition research and other fields. Data and analysis scripts are available at https://osf.io/hktdu/.

**Supplementary Information:**

The online version contains supplementary material available at 10.3758/s13428-021-01724-0.

## Introduction

Social cognition research places great hope in virtual reality (VR) to overcome limitations of laboratory studies and resolve discrepancies between findings obtained within restricted laboratory contexts and naturalistic situations (Parsons et al., 2017; Risko et al., 2012; Rubo & Gamer, 2021; Zaki & Ochsner, 2009). These discussions are based on the critique that social cognition research frequently involves simplified stimuli that do not represent reality, which is multimodal, dynamic, and contextually embedded (Zaki & Ochsner, 2009). One area of research where these considerations became especially prominent in recent years is the field of social attention. In general, attentional shifts towards human beings due to their sole presence in the visual field are well documented (Birmingham et al.,, 2008a,2008b; End & Gamer, 2017; Großekathöfer et al.,, 2020; Rösler et al.,, 2017). However, such preferred visual exploration of conspecifics seems highly reduced in reality (Horn et al., 2021; Laidlaw et al., 2011; Rösler et al., 2021). As a consequence, researchers sought more appropriate research designs that approximate real social environments but at the same time still provide experimental control (Risko et al., 2012; Risko et al., 2016). A solution often discussed in this context is VR, since it allows for multimodal, contextually embedded, and dynamic stimulus presentation (Parsons et al., 2017). In principle, it can enable researchers to observe natural viewing behavior in the laboratory without losing experimental control.

The use of VR for examining social attention is a rather recent development. It has not yet been extensively used to assess attentional prioritization of human beings (for an exception, see Rubo & Gamer, 2021). An experimental design that has been more frequently applied in this domain concerns the examination of social attention in the real world using mobile eye-tracking glasses and comparing these findings to a presentation of video recordings on a computer screen to either the same (Foulsham et al., 2011) or another participant (Rubo et al., 2020). These studies provided initial evidence that attentional allocation towards human beings differs between laboratory and real-life conditions. For example, Foulsham et al., (2011) found generally low fixation probabilities on persons, which were further reduced in real-life conditions when people were near the observer or remained in the visual field for longer durations. Although Rubo et al., (2020) did not confirm a general avoidance of gaze towards conspecifics, they found an increased exploration of people located in the observer’s vicinity. This bias, however, was less pronounced in the real world as compared to the laboratory situation.

Although such studies provide initial evidence for crucial differences between laboratory and field conditions, they are not without limitations. First, participants accomplished different tasks in both contexts (e.g., walking around vs. watching a video), which might induce different patterns of visual exploration (e.g., for avoiding obstacles when planning walking routes). Second, head and body movement were restricted in the laboratory and it is well known that saccadic eye movements differ substantially between conditions with restrained as compared to unrestrained head movements (for a review see Freedman 2008). Third, previous studies involved presenting videos to participants in the laboratory context that were recorded by a head-fixed camera of the same or another participant in the field. Thus, participants in the laboratory could not freely decide where to orient their attention. All in all, these limitations may restrict the generalizability of findings and undermine conclusions that were based on a direct comparison of visual exploration patterns between laboratory and field conditions. Please note that although some of these problems might be addressed by including the video presentation into the real environment itself (Laidlaw et al., 2011), other problems such as the limitation of the field of view (FOV) to the previous recording condition persist. Moreover, such settings might be limited to certain experimental situations where a video playback in the surrounding is not considered unnatural or strange.

In the current study, we designed a novel experimental setting to solve these issues and provide a rich and ecologically valid viewing situation (Shamay-Tsoory and Mendelsohn, 2019; but see Holleman et al.,, 2020 for a critical comment). Specifically, we presented participants with spherical videos^1^ of public places using a head-mounted display (HMD) with an integrated eye tracker. Such stimulation has several advantages compared to previous screen-based experiments. First, it enables participants to actively and freely experience an environment including unrestricted head movements and some degree of body movement (e.g., turning around). Second, the participant’s perspective is contextually embedded in the scene, i.e., she cannot look behind the scene. Whereas in traditional screen-based experiments, participants can evade the stimulation by looking around, such behavior is impossible within the HMD-based presentation of spherical videos. And third, the currently proposed viewing situation enables experimental control over the stimulation, which has been proposed to be one major advantage of VR above field examinations (Parsons et al., 2017).

Compared with 3D rendered virtual scenes, spherical videos come with a number of advantages but also have some limitations. The main advantage is that rich naturalistic stimuli can be generated remarkably faster, cheaper, and easier as compared to the extensive and costly development of 3D worlds. This seems especially true when these scenes include human beings. The main limitations are that interactions with the virtual environment, scenes that hurt physical laws (such as gravity), or scenes with naturalistic 3D properties (i.e., including stereoscopic vision) can hardly be realized with spherical videos. Another challenging aspect for VR in general is movement. Active, self-paced, and continuous movements are difficult, costly, and demanding to include, even in 3D rendered scenes. Since this is basically a form of interaction with the environment, it is impossible to realize with spherical videos. A prominent solution to overcome such problems in 3D rendered scenes is passive movement (e.g., teleportation to a new location), which might also be realized with multiple spherical videos to some degree. After all, the decision on how to realize a VR scene needs careful considerations but it seems plausible to assume that being contextually embedded and empowered to actively experiencing an environment should reduce demand characteristics and elicit a more natural viewing behavior.

The current study aimed at examining the suitability of spherical videos for investigating social attention, and we were interested in better understanding the boundaries of typical laboratory settings. Therefore, we specifically compared visual exploration patterns of participants when viewing spherical videos of five public places in the laboratory to their behavior when visiting the same spots in the real world. We chose to examine participants’ behavior at several locations in order to ensure generalizability of findings across situational characteristics and to permit assessing the reliability of the current method by estimating the consistency of viewing patterns across the different locations in the video as well as the real-life condition. Moreover, we specifically compared viewing behavior between conditions to determine to what degree measures of social attention generalize from the laboratory to field contexts. Although we are convinced that the currently used spherical videos have some advantages over previously used stimulation conditions, they still differ from the real world since participants cannot socially interact with pedestrians in the video and do not have to follow certain social norms when viewing the scenes in the laboratory (e.g., staring will not have consequences, Ellsworth et al., 1972). Since both factors are suspected of playing a critical role in attentional allocation towards conspecifics (Foulsham et al.,, 2010; Laidlaw et al.,, 2011; see also, Gobel et al.,, 2015for a discussion on the dual function of gaze), we expected a reduced amount of social attention in the real world as compared to the viewing of spherical videos. Finally, for exploratory purposes, we related the currently observed viewing behavior to questionnaire data on autistic personality and social anxiety traits.

## Methods

Hypotheses, sample size, design specifications, and analysis steps were preregistered before data collection on Aspredicted.org (available at: https://aspredicted.org/p7a83.pdf). In our study, we used a fully nested within-subjects design with the factors environment (virtual environment vs. real environment) and region of interest (ROI, person vs. object, see below for further details).

### Participants

The sample consisted of 44 participants (33 female; age: *M* = 22.10 years; *SD* = 6.00 years) who were recruited via the online participant pool of the University of Würzburg. Students participated for course credit. All participants had normal or corrected-to-normal vision by means of contact lenses. Sample size planning was done using PANGEA (Westfall, 2016) before collecting any data^2^. The planned sample size allows for detecting the anticipated interaction of interest with a medium effect (Cohens *d* = 0.3) at a conventional level of *α* = .05 and an adequate power of 1 - *β* = 0.87.

### Stimuli and apparatus

**Fig. 1:**
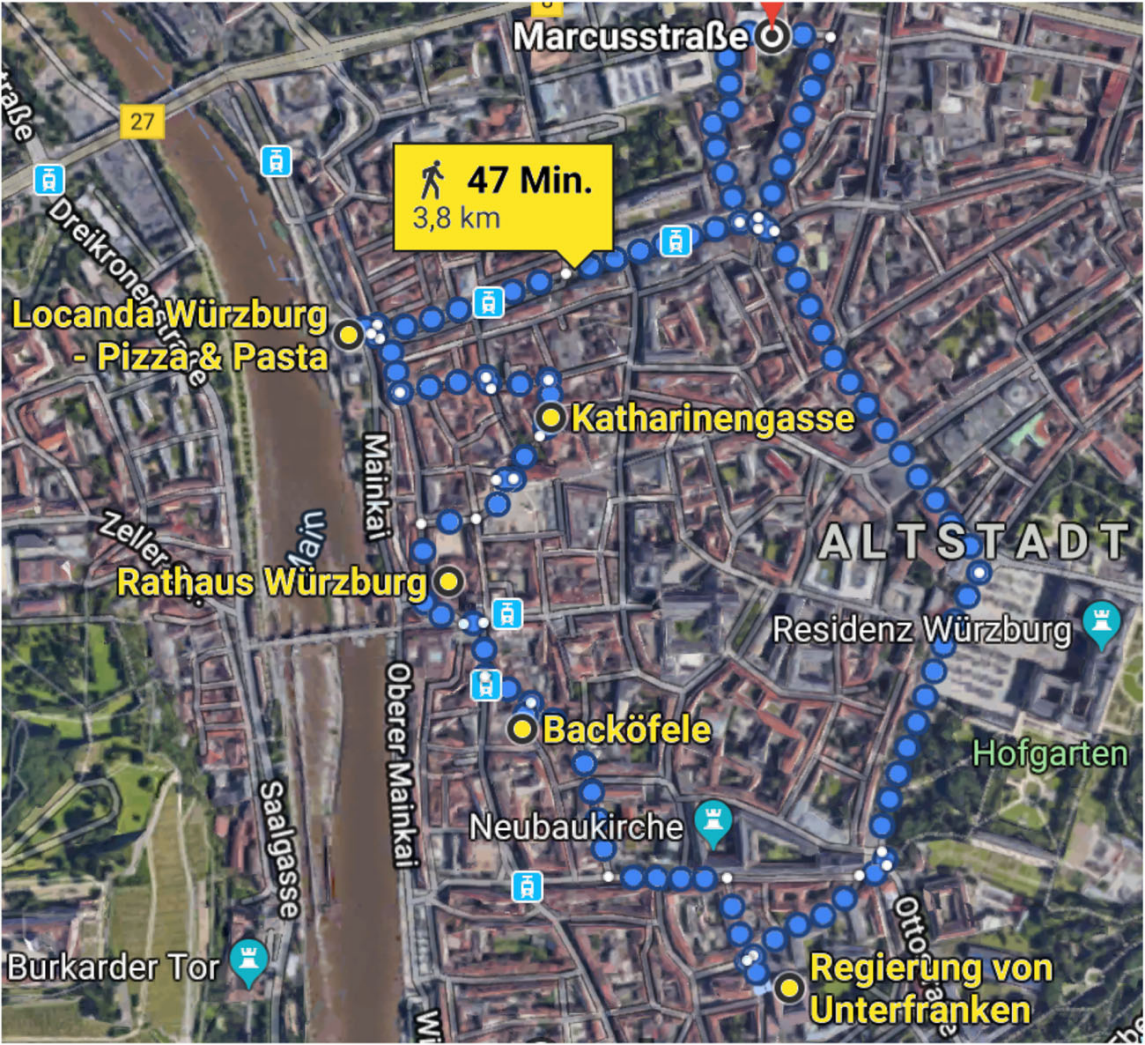
Google Maps route the experimenter and participants walked to the five measurement locations (highlighted with *yellow dots* and corresponding descriptive labels) in the real environment. Spherical videos were recorded at the identical locations for the virtual environment

The eye-tracking data were collected for five different locations in Würzburg, Germany. The participants experienced the selected locations in two environments: in the virtual environment (VE) through watching spherical videos in an HMD and in the real environment (RE) by visiting the location in reality (see Fig. 1).

#### Locations

The five locations in the city of Würzburg included places located in rather quite side streets as well as more crowded spots. On average, the number of pedestrians was comparable between the VE (*M* = 10.60, *SD* = 6.23) and the RE (*M* = 8.18, *SD* = 6.15).

Locations were visited in RE at the shortest route to keep walking time minimal. During the experiment, the route was used in two different directions, counterbalanced across participants. The order of locations in both environments was kept identical for each participant, resulting in only two sequences of spherical videos in the VE.

#### Virtual environment

The stimuli for the VE were spherical videos recorded at the five locations with a GoPro Omni camera mount of six *GoPro HERO4 black* cameras. The six resulting videos were then stitched together into a single spherical video for each location using *Kolor Autopano Pro* (Version 4.2). The final video had a total resolution of 3840 × 1920 pixels with 50 frames per second (FPS) and a duration of 15 s^3^. We added an additional seven seconds of black screen (5 s at the start and 2 s at the end) and the audio track of one camera to each video.

Two videos from each location were used for each participant resulting in 30 s of spherical videos per location. The videos were projected on a virtual sphere rendered by the 3D game engine Unity (Version 2018.2.18f1) onto an HTC Vive. We used the HMD with HTC Vive’s default internal rendering resolution of 3024 × 1680 pixels (or 1512 × 1680 pixels per eye and display) at a refresh rate of 90 FPS. The HTC Vive provides a FOV of approximately 110^∘^ × 110^∘^ of visual angle at a typical distance of 10 mm from the eyes to the internal displays. Eye-tracking data relative to the FOV were collected with an integrated SensoMotoric Instruments (SMI) binocular eye tracker and the SMI plug-in for Unity at a sampling rate of 250 Hz.

#### Real environment

Eye tracking in the RE was conducted using SMI Eye-Tracking Glasses 2.1 with the iViewETG software at a sampling rate of 60 Hz. The integrated camera recorded the participants’ FOV at 30 Hz with a resolution of 1280 × 960 pixels. The FOV amounts to approximately 60^∘^ × 46^∘^ of visual angle.

### Procedure

Upon arrival, participants were welcomed and provided written informed consent. To conceal the aim of the current study and ensure that participants are not concentrating on their own eye movements, the experimenter provided erroneous information that we were interested in examining the suitability of the current devices for measuring pupil width in different environments. Following the general introduction, the participants started with one of the environments. The starting environment was counterbalanced between participants as well as the specific route they walked or the sequence of the spherical videos they watched, respectively.^4^ Consequentially, measurements in the RE were conducted directly at the five locations in Würzburg and in the VE, measurements took place in a laboratory of the University of Würzburg.

#### Virtual environment

For the virtual environment, we first equipped and positioned participants with the HMD and headphones in the laboratory. Before we started the sequence of spherical videos, we asked participants to accomplish the numerical validation as provided by the manufacturer SMI as well as an external three-point validation (the average distance between validation marks and the recorded gaze points amounted to *M* = 1.91^∘^, *SD* = 1.42^∘^). Afterwards, participants started watching the spherical videos while being able to actively explore the environment with unconstrained head and eye movements. Furthermore, participants were allowed to move their body (e.g., to turn around) but they were instructed not to walk. After all spherical videos were played, we repeated the initial validation procedure, to ensure that the device was still properly calibrated (deviation between validation marks and gaze coordinates: *M* = 1.28^∘^, *SD* = 0.66^∘^). Directly after the exposure we assessed presence, i.e., the feeling of being there in a VR using the Igroup Presence Questionnaire (Schubert, 2003). Participants indicated a moderate feeling of presence (*M* = 3.89, *SD* = 0.89) on a scale 0 to 6. Simulator sickness was assessed using the Simulator Sickness Questionnaire (SSQ, Kennedy, Lane, Berbaum, & Lilienthal, 1993). Participants reported absence of most sickness symptoms and reached a total score of *M* = 27.97 (*SD* = 21.36) on a scale ranging from 0 to 235.62.

#### Real environment

For the real environments, we equipped participants with mobile eye-tracking glasses. Additionally, we asked participants to wear a baseball cap to reduce the influence of direct sunlight. Before walking to the first location in the real environment, the eye tracker was calibrated and validated in the laboratory using a three-point validation procedure (average distance between validation marks and the recorded gaze points amounted to *M* = 2.65^∘^, *SD* = 2.93^∘^). Then the experimenter walked with the participant to the first location of one of the two predetermined routes. At every location, the eye tracker was again calibrated using three predetermined landmarks. After calibration, participants received the instruction to hold a notebook for about 10 s in front of their face and thus cover the camera of the eye tracker. This was required to further align recording conditions between virtual and real environments: It simulated a sudden trial onset and reduced the influence of prior contextual information similar to the VE. On top, it was also used as an objective starting point for data analysis (see Image data processing below). Participants were further told that the experimenter would move out of their sight and were shown the direction of the hide-out. After answering potential questions of the participant, the experimenter asked them to bring the notebook in position and moved away. Participants were given about 2 min to freely explore the environment before the experimenter reentered the FOV and ended the trial. Since the experimenter had no further control over the behavior of the participant when waiting in the hide-out (e.g., about the precise time point when exploration of the surrounding started), we deliberately chose a longer viewing time than in the VE to ensure a sufficient amount of usable data. Note that during active exploration of the environment, participants were not allowed to walk to keep the situation as similar to the VE as possible. During the recording, the experimenter tried to overview the location from her hide-out and estimated the number of pedestrians around the participant. For crowded places where the experimenter lost track of the total number of pedestrians, we set an upper limit of 20 pedestrians. Afterwards, the experimenter accompanied the participant to the next location, and the procedure was repeated. After the last location, the experimenter and the participant returned to the laboratory where the eye tracker was calibrated and validated once more to ensure that proper recording quality could still be achieved (deviation between validation marks and gaze coordinates: *M* = 2.16^∘^, *SD* = 1.79^∘^).

### Questionnaires

After finishing measurements in both environments, we asked participants to complete a brief demographic questionnaire, the Social Interaction Anxiety Scale (SIAS, Stangier, Heidenreich, Berardi, Golbs, & Hoyer, 1999, *M* = 20.30, *SD* = 8.57, *Range* = 8 to 41) and the Autism-Spectrum-Quotient short version (AQ-k, Freitag et al., 2007, *M* = 6.50, *SD* = 3.32, *Range* = 1 to 17). Upon completion of the questionnaires, we disclosed the actual aim of the study and explained that we measured gaze positions instead of pupil responses. We then offered the opportunity to delete the participants’ data upon request but no participant made use of this possibility.

### Image data processing

To analyze participants’ viewing behavior in both environments, we manually scored what participants were looking at in their FOV. We tried to align data processing and analysis for both environments as closely as possible to permit a direct comparison of gaze patterns between viewing conditions. For that purpose, we first extracted individual video frames from both environments. For image data processing, we used Python (Version 3.7, Van Rossum & Drake, 1995) with OpenCV (Heinisuo, 2019), NumPy (Oliphant, 2019) and Pandas (The PyData Development Team, 2019). For the VE, frames were extracted directly using Unity’s screenshot function at 5 FPS at half of the internal monocular rendering resolution of the HMD (i.e., 756 × 840 pixels) resulting in a total of 150 frames for each location. For the RE, we first exported a video of the FOV as well as the log file for each participant and location via the software BeGaze (Version 3.7, SMI, 2017). Next, we extracted all frames at 5 FPS from the videos using OpenCV (Heinisuo, 2019). We kept 150 frames from trial onset to cover a comparable time window as for the VE. Trial onset was exactly 15 frames, i.e., 3 s after the notebook vanished from the first of all extracted frames. The delay of 3 s was necessary for the camera to adapt to the sudden change in lighting conditions due to the removal of the notebook. The resulting frames had a resolution of 1280 × 960 pixels. To prepare the extracted video frames for manual scoring, we added a gaze point at the respective gaze coordinates in form of a circle with a size of 1^∘^ to the video frames using OpenCV (Heinisuo, 2019). Accordingly, the frame of reference for the gaze coordinates can be classified as head-centered (for more details and a discussion on the use and terminology of frame of references in eye-tracking research see Hessels, Niehorster, Nyström, Andersson, & Hooge, 2018). These gaze coordinates resembled the binocular gaze from a hypothetical cyclopic eye, as internally processed by the SMI software. Subsequently, two raters categorized the gaze points on a total of 66,000 video frames (44 participants × 2 conditions × 5 locations × 150 frames). Each rater scored one half of the stimulus set using the following scheme: First, raters categorized whether other persons were present in a given frame. For frames with persons present, raters additionally scored the gaze point as falling on one of three ROIs (*person*, *object*, *background*). Scoring followed a hierarchical assignment. If any part of the gaze point overlapped with any part of a person, the ROI for the frame was scored as *person*. If the gaze point was not scored as *person* but overlapped with an object that could be directly interacted with (e.g., car, bike, sign, baby carriage), it was scored as *object*. If the ROI was neither scored *person* nor *object* the gaze point was scored as *background* (e.g., sky, crosswalk, buildings). Frames missing a gaze point (e.g., due to blinks or recording difficulties) were categorized as *missing gaze*. For the analyses, we excluded all frames with *missing gaze* and frames in which no persons were present. To ensure that raters were consistent in their scoring, a subset of five participants (i.e., 7500 frames) was scored by both raters, and we assessed their interrater reliability. Cohen’s *κ* = .87 indicated a good agreement between both raters.

In VE, 97% all frames included valid gaze points, from which 73% of frames were further analyzed based on the presence of persons. In RE in contrast, valid measures of gaze points were only present in 58% of frames, from which again 73% included persons. Thus, the final analyses were based on 71% and 42% of data from the VE and RE, respectively.

### Data analysis

We used the programming language R (version 3.6, R Core Team, 2020) for statistical analyses and numerical data processing. Specifically, we relied on the functionality provided by the *tidyverse* packages (Wickham, 2017) for data processing. To conduct linear mixed models, we used the *afex* package (Singmann, Bolker, Westfall, Aust, & Ben-Shachar, 2019) as an interface for *lme4* functions (Bates, Mächler, Bolker, & Walker, 2015). Degrees of freedom to calculate *p* values from the according *t*-distribution for the linear mixed model were obtained using the Sattertwhaite approximation (with *afex* via *lmerTest* package, Kuznetsova, Brockhoff, & Christensen, 2017). To calculate and plot the models’ estimated marginal means, we used the *emmeans* package (Lenth, 2020). We used the conventional threshold of *α* = .05 for determining statistical significance. All analysis scripts and data are available at https://osf.io/hktdu/.

#### Confirmatory analysis

To test the main hypothesis that social attention differs between VE and RE, we calculated the average gaze proportion on each ROI as a function of the environment for each participant and conducted a linear mixed model on these proportions using the fixed effects environment and ROI (ROI: persons or objects). Please note that the background ROI was dropped since all proportions sum up to 1 and thus the background information is redundant. The random effect structure for this Model 1 included random intercepts for participant ID and followed the preregistered *a-priori restricted model*. Although it would have been possible to also include a random intercept for location, we decided to rather rely on a parsimonious account and kept the preregistered model simple but suitable to address our research question. This approach seemed adequate given the small number of locations (Judd, Westfall, & Kenny, 2012) and it followed conventions used in the field (i.e., 2 × 2 ANOVA designs on data aggregated across trials) as well as considerations that the variance-covariance matrices could be estimated precisely enough to avoid singularity (Matuschek, Kliegl, Vasishth, Baayen, & Bates, 2017).

Additional models were built upon the preregistered model but now included a maximum random effect structure with respect to the newly added predictors as recommended by Barr, Levy, Scheepers, and Tily (2013). First, we added predictors for *social anxiety* (Model 2) and *autism spectrum* traits (Model 3) to Model 1. For both new models, we included all additional two-way interactions as well as the three-way interaction of all factors. We used sum-to-zero contrasts for categorical factors in all models. To test the performance of the resulting models, we compared the log-likelihood of Models 2 and 3 to the preregistered Model 1.

To analyze the consistency of viewing behavior across the five locations in each environment, we calculated Cronbach’s *α* of gaze proportions using the *psych* package (Revelle, 2019). The generalizability across both environments was assessed by correlating average viewing preferences between VE and RE. Finally, to estimate the stability of viewing patterns between identical locations viewed in VE and RE, we calculated correlations between gaze proportions at each location and pooled them using Fisher *z*-transformations. All these analyses were accomplished separately for visual exploration of persons and objects, respectively, and the whole pattern of correlations was visualized using a correlation matrix including all pairwise Pearson correlation coefficients *r* for gaze proportion at each location in each environment for each ROI.

#### Exploratory analysis

For exploratory purposes, we conducted an additional linear mixed model (Model 4) including the number of pedestrians at the locations as a continuous fixed effect (*min* = 1, *max* = 20, standardized to *M* = 0 and *SD* = 1) and location as an additional random effect. Again, we initially specified the full random effects structure as in Models 2 and 3. As the full model did not converge, we pruned the model stepwise which resulted in a restricted model that included only uncorrelated random slopes for locations.

Finally, in order to elucidate general differences in visual exploration behavior between RE and VE, for example regarding the center bias relative to the FOV (Tatler, 2007), we plotted a smoothed density map (Gaussian kernel with a standard deviation of 1^∘^ of visual angle) of gaze positions relative to the FOV for a central viewing region spanning 60^∘^ × 46^∘^ for both environments across all participants.

## Results

### Comparison of social attention between real and virtual environment

**Fig. 2:**
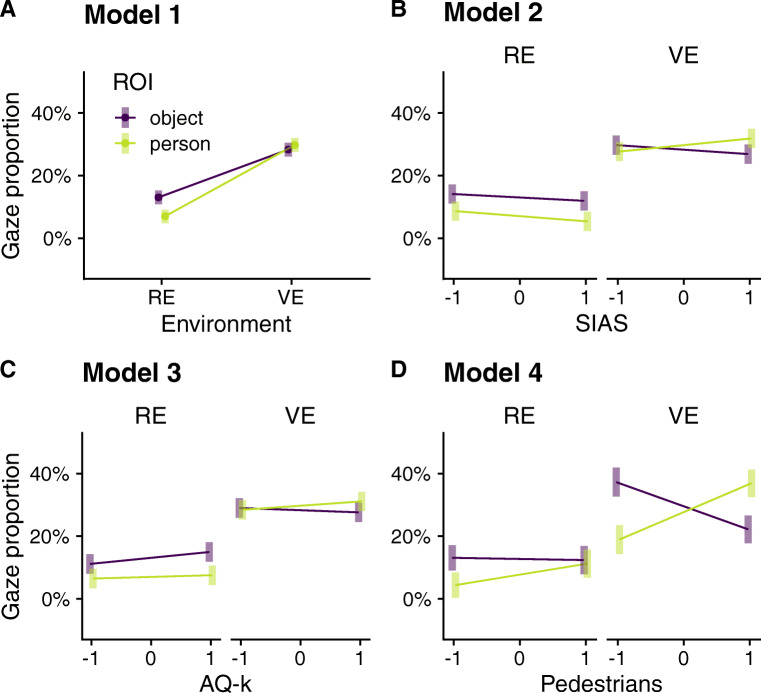
Interaction plots of all linear mixed models showing estimated marginal means as a function of the included predictors. *Error bars* depict 95% confidence intervals of predicted values. (A) The preregistered Model 1 included environment (real environment, RE vs. virtual environment, VE) and ROI (person vs. object) as predictors. Models 2 (B) and 3 (C) additionally included standardized scores of the Social Interaction Anxiety Scale (SIAS, depicted range from 12 (-1 *SD*) to 29 (+ 1 *SD*)), and the Autism-Spectrum-Quotient short version (AQ-k, depicted range from 3 (-1 *SD*) to 10 (+ 1 *SD*)), respectively. (D) Compared to Model 1, Model 4 additionally included the standardized number of pedestrians in the environment at each location (depicted range from 3 (-1 *SD*) to 15 (+ 1 *SD*)).

To test our main hypothesis, we conducted the preregistered linear mixed model on gaze proportions with environment (RE vs. VE) and ROI (person vs. object) as fixed effects and participant ID as random effect.^5^ This analysis revealed significant main effects for environment and ROI that were qualified by significant interaction of both factors (see Table 1). Overall, participants tended to look more on objects than on persons, but the significant interaction effect indicates that this was only true for the RE (*M*_RE,object_ = 0.13, *SD*_RE,object_ = 0.05, *M*_RE,person_ = 0.07, *SD*_RE,person_ = 0.06, *t*(129) = 4.03, *p* = .001) but not for the VE (*M*_VE,object_ = 0.28, *SD*_VE,object_ = 0.05, *M*_VE,person_ = 0.30, *SD*_VE,person_ = 0.11, *t*(129) = -0.99, *p* = .758). These findings confirm our primary hypothesis that social attention is reduced in the real world. Furthermore, the main effect of environment describes a general tendency of fewer gazes on persons and objects - and thus an increased amount of background exploration - in the RE as compared to the VE (see Fig. 2A).

**Table 1:** Estimated coefficients for the preregistered Model 1 with environment and ROI as fixed and participant ID as random effects for the prediction of gaze proportions

	Estimate	*SE*	*Df*	*t*	*p*
Intercept	0.20	0.01	43	31.00	<.001
Environment (RE)	− 0.10	0.01	129	-18.01	<.001
ROI (object)	0.01	0.01	129	2.15	.033
Environment (RE) × ROI (object)	0.02	0.01	129	3.55	.001

The linear mixed model is based on sum-to-zero contrasts. *RE* real environment, *ROI* region of interest

### Consistency of viewing behavior within and across environments

**Fig. 3:**
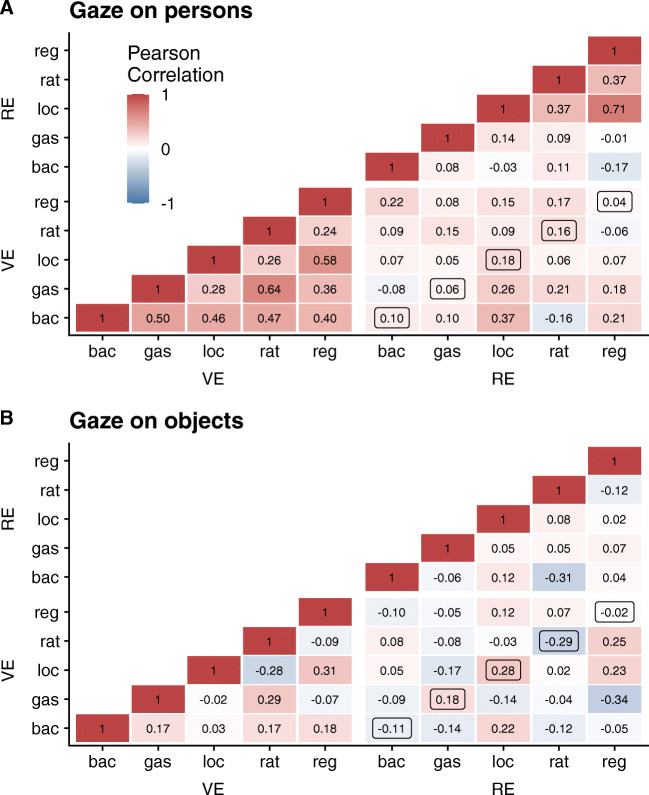
Heat map of pairwise Pearson correlation coefficients *r* for gaze on persons (A) and objects (B) for all five locations in both environments. Framed coefficients highlight correlations at identical locations between both environments.

In a first step, we assessed the consistency of gaze proportions on persons and objects, respectively, within each environment. Figure 3A illustrates that gaze on persons was more stable across locations in the VE (lower left triangle) as compared to the RE (upper right triangle). This difference was also evident in measures of internal consistency, which were substantially higher for the VE (Cronbach’s *α* = .75, 95% CI [.64, .86]) compared to RE (Cronbach’s *α* = .38, 95% CI [.32, .44]). By contrast, no such consistency was evident in gaze on objects (see Fig. 3B) and we obtained low values of Cronbach’s *α* in both, the VE (Cronbach’s *α* = .29, 95% CI [.24, .34]) and the RE (Cronbach’s *α* = -.03, 95% CI [-.08, .03]). In order to estimate the generalizability of viewing patterns across VE and RE, we first calculated the correlation between average gaze proportions across locations between both environments. Although the correlation was positive for gaze proportions on persons (*r* = .22, 95% CI [−.08, .48], *t*(42) = 1.46, *p* = .153) but close to 0 for objects (*r* = .01, 95% CI [−.29, .30], *t*(42) = 0.04, *p* = .965), both correlations were not statistically significant and 95% confidence intervals overlapped. In a second step, we only focused on the correlation of gaze proportions between identical locations in the VE and the RE (see the highlighted diagonal in the lower right of Fig. 3A and B). Although the average correlation was again descriptively higher for gaze on persons (*r* = .11) than on objects (*r* = .01), values are generally low, which indicates that viewing behavior differed between environments.

**Fig. 4:**
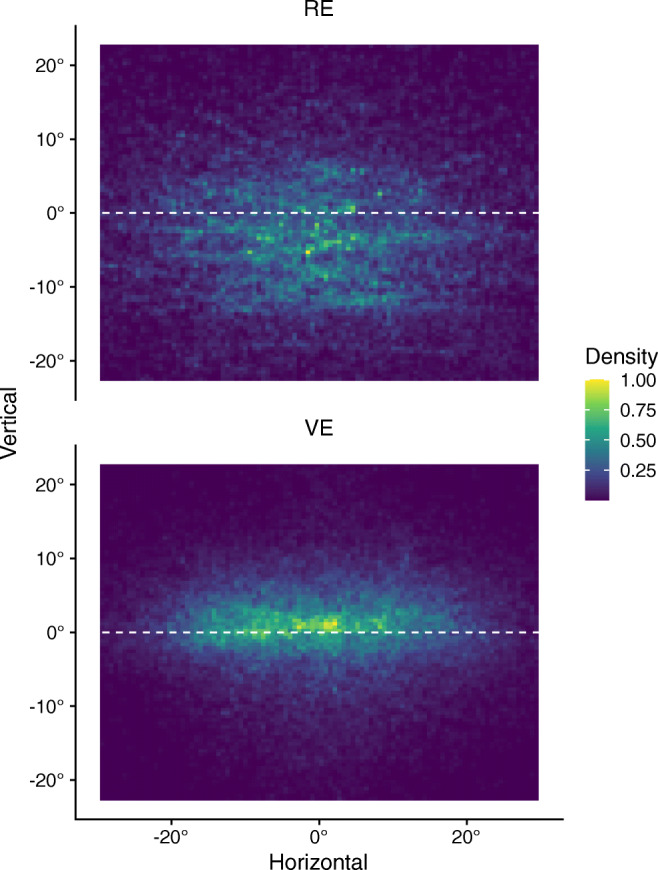
Smoothed density maps illustrating the distribution of gaze coordinate relative to the field of view in the real environment (RE) and the virtual environment (VE). Densities are depicted for a central area of 46^∘^ vertically and 60^∘^ horizontally (normalized to a range of 0 to 1).

The spatial distribution of gaze coordinates within the FOV also indicates strong differences between VE and RE (see Fig. 4). Whereas gaze points mostly clustered below the horizon in the RE and showed a larger spread on the vertical axis, they were vertically more centered slightly above the horizon in the VE.

### Influence of personality traits

**Fig. 5:**
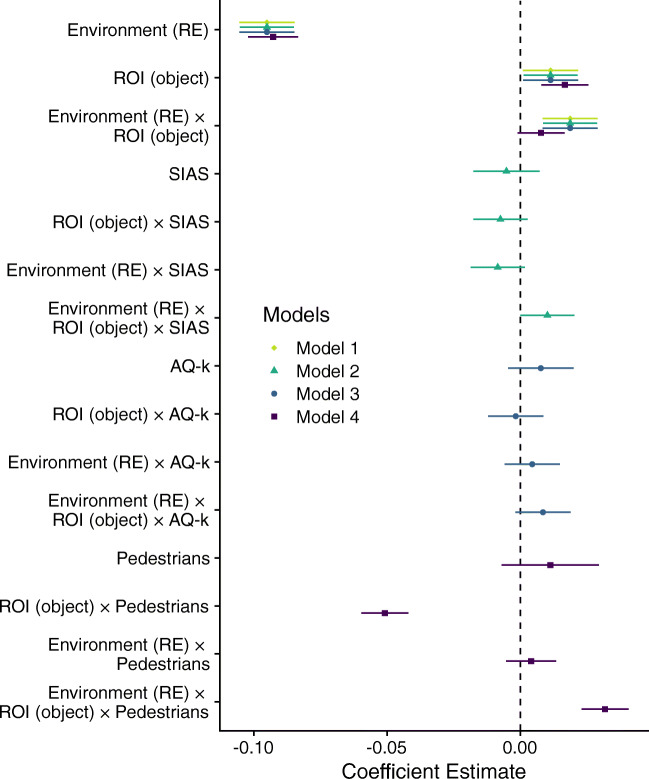
Dotwhisker plot of estimated model coefficients for Model 1 to 4 (*dots*) with 95% confidence intervals shown as whiskers. Environment (RE): Environment reference *Real environments*, ROI (object): ROI reference *Objects*, SIAS: standardized sum score of the Social Interaction Anxiety Scale, AQ-k: standardized sum score of the Autism-Spectrum-Quotient short version. Pedestrians: standardized number of pedestrians.

To exploratively test the influence of relevant personality traits on viewing patterns, we separately extended our preregistered Model 1 with the standardized scores of the SIAS (Stangier et al., 1999) and the AQ-k (Freitag et al., 2007) as fixed factors. The linear mixed model conducted to examine the influence of social anxiety (Model 2) did supply only weak evidence that social anxiety influences gaze proportions. Specifically, the three-way interaction between ROI, environment, and social anxiety just failed statistical significance (see Table 2). Interestingly, the previously estimated coefficients were very robust and did not change substantially with the inclusion of SIAS scores (see Fig. 5). The linear mixed model incorporating autistic traits (Model 3) showed the same weak influence on gaze proportions (see Table 3). Again, previously observed effects were very robust (Figure 5).

**Table 2:** Estimated coefficients for the Model 2 with environment, ROI and SIAS as fixed and participant ID as random effects for the prediction of gaze proportions

	Estimate	*SE*	*Df*	*t*	*p*
Intercept	0.20	0.01	42	30.88	<.001
Environment (RE)	− 0.10	0.01	126	− 18.40	<.001
ROI (object)	0.01	0.01	126	2.20	.030
SIAS	− 0.01	0.01	42	− 0.82	.418
Environment (RE) × ROI (object)	0.02	0.01	126	3.62	<.001
Environment (RE) × SIAS	− 0.01	0.01	126	− 1.44	.153
ROI (object) × SIAS	− 0.01	0.01	126	− 1.63	.105
Environment (RE) × ROI (object) × SIAS	0.01	0.01	126	1.97	.052

The linear mixed model is based on sum-to-zero contrasts. *RE* real environment, *ROI* region of interest, *SIAS* standardized sum score of the Social Interaction Anxiety Scale

**Table 3:** Estimated coefficients for the Model 3 with environment, ROI and AQ-k as fixed and participant ID as random effects for the prediction of gaze proportions

	Estimate	*SE*	*Df*	*t*	*p*
Intercept	0.20	0.01	42	31.18	<.001
Environment (RE)	− 0.10	0.01	126	− 18.04	<.001
ROI (object)	0.01	0.01	126	2.16	.033
AQ−k	0.01	0.01	42	1.23	.226
Environment (RE) × ROI (object)	0.02	0.01	126	3.55	.001
Environment (RE) × AQ−k	0.00	0.01	126	− 0.32	.748
ROI (object) × AQ−k	0.00	0.01	126	0.85	.398
Environment (RE) × ROI (object) × AQ−k	0.01	0.01	126	1.61	.110

The linear mixed model is based on sum-to-zero contrasts. *RE* real environment, *ROI* region of interest, *AQ-k* standardized sum score of the Autism-Spectrum-Quotient short version

Ratio log-likelihood tests between our preregistered model and the additional models considering individual differences in social anxiety and autistic traits supported our impression that model performance did not benefit from including personality factors. The additional parameters in the two latter models did not significantly increase model performance (see Table 4).

**Table 4:** Model comparison of the preregistered model with the models including personality traits

	Coefficients	AIC	BIC	LogLik	deviance	*chi*	*Df*	*p*
Model 1	6	− 412.83	− 393.81	212.42	− 424.83			
Model 1 - Model 2	10	− 414.23	− 382.52	217.11	− 434.23	9.4	4	.052
Model 1 - Model 3	10	− 409.91	− 378.21	214.96	− 429.91	5.08	4	.279

*AIC* Akaike information criterion; *BIC* Bayesian information criterion; *Df* Residual degrees of freedom

### Relevance of the number of persons present in a scene

In general, the average number of pedestrians was comparable between the VE (*M* = 10.60, *SD* = 6.23) and the RE (*M* = 8.18, *SD* = 6.15), but there was substantial variability between locations, both in the RE (*Min* = 4, *Max* = 20) as well as the VE (*Min* = 4.40, *Max* = 19.07). In an additional exploratory analysis, we examined to what degree this number of persons who were present at a given location affects viewing behavior. We therefore added the number of pedestrians to the previously specified Model 1. This value was constant for every video shown in the VE but was estimated individually by the experimenter in RE. The newly specified linear mixed Model 4 included locations as an additional random effect and the number of pedestrians in the environment as an additional fixed effect, plus all interaction terms. The maximum model, including random intercepts for location and random slopes for the number of pedestrians at each location, did not converge. Therefore, we estimated the model suppressing the correlations between the random intercepts for location and random slopes for pedestrians^6^. Most interestingly, the two-way interaction between environment and ROI was substantially reduced in this model and did not remain statistically significant (see Table 5 and Fig. 5). This was probably due to the strong three-way interaction between ROI, environment, and the number of pedestrians. Figure 2B shows that in the VE, a high number of pedestrians was associated with enhanced gaze on persons as compared to objects, but this pattern flipped when only a few people were around. Qualitatively, such a pattern was also evident in the RE, but it was much less pronounced, and gaze proportions on persons never exceeded gaze proportions on objects.

**Table 5:** Estimated coefficients for the Model 4 with environment, ROI and number of pedestrians as fixed and participant ID and location as random effects for the prediction of gaze proportions

	Estimate	*SE*	*Df*	*t*	*p*
Intercept	0.20	0.01	4.84	14.08	<.001
Environment (RE)	− 0.09	0.00	129.67	− 19.43	<.001
ROI (object)	0.02	0.00	811.74	3.73	<.001
Pedestrians	0.01	0.01	2.26	1.21	.337
Environment (RE) × ROI (object)	0.01	0.00	811.74	1.74	.082
Environment (RE) × Pedestrians	− 0.05	0.00	811.74	− 11.26	<.001
ROI (object) × Pedestrians	0.00	0.00	545.23	0.85	.394
Environment (RE) × ROI (object) × Pedestrians	0.03	0.00	811.74	7.05	<.001

Model 4: The linear mixed model is based on sum-to-zero contrasts. *RE* real environment, *ROI* region of interest. The number of pedestrians was included as a standardized value with *M* = 0 and *SD* = 1. Location was included as additional random effect including uncorrelated random intercepts and random slopes by pedestrians

## Discussion

In the current study, we directly compared viewing behavior in real and virtual environments with a specific focus on social attention using spherical videos as a novel stimulation technique. In general, our results support previous findings (Foulsham et al., 2011; Laidlaw et al., 2011; Rubo et al., 2020) of a reduced attention towards conspecifics in the real as compared to the virtual environment. Extending previous studies, these results were obtained even when closely matching the laboratory environment to reality by using spherical videos recorded at the same locations that were also visited in the real world. These conditions allowed participants to freely explore and actively experience naturalistic stimuli in the laboratory while being contextually embedded in the environment. Since we observed reduced social attention in the real environment even in such closely matched conditions and a low correlation of gaze proportions on persons between both environments, our results indicate that the possibility to socially interact with other persons is the main driver of these differences between conditions. It thus seems sensible to assume that a real confrontation with conspecifics enhances the activation of social norms (e.g., not staring at others) and thus results in a reduced overt visual exploration of other persons in real life. This hypothesis is also supported by the observed modulation of this effect by the number of pedestrians in the surroundings. Whereas gaze on other individuals increased strongly with the number of pedestrians in the virtual environment, this effect was substantially weaker in the real world. Collectively, these findings indicate that it is not sufficient to focus on aspects of the viewing situation (e.g., active exploration, contextual embedding) to enhance the generalizability of laboratory findings on social attention to the real world. The main aspect that modulates attention towards conspecifics seems to be the actual presence of other persons and the associated possibility for an interaction (cf. Zaki & Ochsner, 2009; Risko et al., 2016). These findings call for an enhanced focus on social interactions in social cognition research (Jaegher, Paolo, & Gallagher, 2010).

In addition to these variations of social attention between real and virtual environments, we also observed more general differences in viewing behavior between contexts. Interestingly, attention towards conspecifics seems to be more stable across locations in the virtual than the real environment and measures correlated only weakly between conditions. This could indicate that attentional preferences that were recently described for several semantic features and visual properties (de Haas, Iakovidis, Schwarzkopf, & Gegenfurtner, 2019; Linka & de Haas, 2020; Rubo & Gamer, 2018) are more robust in laboratory than in real-life conditions and do not necessarily generalize from the laboratory to field contexts. Regarding gaze on objects, we neither found a stability of gaze proportions within each environment nor between conditions but this finding might also be attributed to the rather broad categorization of objects that neglected specific object classes (e.g., cars, symbols, text) or dimensions (e.g., static vs. moving or artificial vs. natural objects).

We also observed general differences in the spatial distribution of gaze coordinates within the FOV between virtual and real environments (see Fig. 4). In both cases, a center bias (Tatler, 2007) was evident which is consistent with previous research using mobile eye-tracking in the field (Foulsham et al., 2011, Ioannidou, Hermens, & Hodgson, 2016) and stationary eye-tracking during video viewing (e.g., Tseng, Carmi, Cameron, Munoz, & Itti, 2009). However, this center bias was much more pronounced in the virtual environment where participants showed a substantially reduced spread of gaze points along the vertical axis. The reasons for this discrepancy remain elusive. On the one hand, it might be related to the HMD itself since wearing such device was novel to most participants (only 7% of the current sample indicated some previous experience with virtual reality). On the other hand, it could also result from an interaction between head and eye movements (Einhäuser et al., 2007) since participants were free to move their head in both environments. Unfortunately, tracking head movements could not be accomplished with the currently used eye-tracking glasses, which precludes a detailed analysis of differences between conditions. Thus, it remains unclear whether participants more strongly relied on head movements to visually explore their surroundings in the virtual environment or whether the observed enhanced center bias in this condition indeed reflects less exploration. Furthermore, in the real environment, gaze was more concentrated below a relative horizon. Interestingly, this is compatible with results from studies with walking participants (e.g., Foulsham et al., 2011 or Matthis, Yates, & Hayhoe, 2018) even though participants were not allowed to walk in the current study. Although speculative, this could indicate that the real environment primed participants to engage in a more active mode of visual exploration that includes planning for potential walking movements. Taken together, these general differences between viewing conditions highlight the need for future studies to elucidate these aspects in more detail before uncritically translating experimental paradigms to VR environments and assuming comparability to field conditions.

Regarding the influence of personality traits on gaze proportions, we neither observed significant effects of social anxiety nor of autism spectrum traits. This contrasts with previous studies that documented reduced attention towards faces or eyes of conspecifics in individuals with high autism spectrum (Hessels, Cornelissen, Hooge, & Kemner, 2017, Laidlaw et al., 2011) or social anxiety traits (Howell, Zibulsky, Srivastav, & Weeks, 2015, Rubo et al., 2020), respectively. Note, however, that some studies did not observe general effects of such traits but rather only for specific situations, e.g., an effect of social anxiety on gaze at people in the vicinity of the observer (Rubo et al., 2020). Moreover, other studies failed to observe effects of social anxiety or autism spectrum traits on measures of social attention in real environments (e.g., Rösler et al., 2021; Horn et al., 2021; Vabalas & Freeth, 2015). The current findings might therefore be attributed to a genuine absence or a very small effect of personality traits on viewing patterns, which could not be reliably detected with the limited sample size of the current experiment. Alternatively, such effects might only surface in more heterogeneous samples that also include participants with clinically relevant autism spectrum or social anxiety symptoms.

Although our study has several strengths including a close matching of laboratory and field conditions regarding data acquisition and analysis, it also comes with some limitations. First, scene presentation in the laboratory was somewhat restricted by technical limitations of the HMD. For example, the display resolution degraded the degree of detail of objects and pedestrians in the distance. However, we do not believe that these limitations had a major impact on the results of this study since the videos were short, novel and interesting and therefore effectively captured participants’ attention. No participant complained about the presentation quality or spontaneously mentioned problems with the HMD. We believe that these technical limitations will also become weaker as this technology matures. Second, most of the participants were not experienced with VR and this novelty might lead to certain viewing biases. However, as the current results are comparable with previous findings obtained in other settings (Foulsham et al., 2011; Rubo et al., 2020) and since we observed more consistent instead of more variable viewing patterns in the virtual environment, we suspect these biases to be rather small. Third, our research design involved walking to the locations in the real environment and consequently, participants had prior information about the location before the actual trial began. This difference to the VE could hardly be eliminated but we tried to reduce its impact by choosing well-known locations in the city of Würzburg, Germany, that should be familiar to most participants. Moreover, to align recordings conditions between virtual and real environment, we required participants to use a notebook to cover their sight before starting measurements in the RE. This procedure was implemented to reduce the influence of contextual information and to simulate a sudden trial onset similar to the VE. Fourth, although we tried to match presentation conditions in virtual and real environments as closely as possible, some environmental factors were beyond experimental control. Apart from weather conditions and daytime, this mainly applied to the number and behavior of pedestrians at the different locations. However, the average number of pedestrians was comparable between both environments and we explicitly considered the variability across locations in an exploratory statistical analysis that also revealed a crucial influence of this factor on measures of social attention. Fifth, reality is multimodal. Within our setup, we tried to account for this by including visual and auditory stimulation in the virtual environment (Zaki & Ochsner, 2009). Although we suggest that these two modalities are most important for generating a sense of presence, it seems interesting for future research to stimulate additional senses (e.g., olfaction) and improve the audiovisual stimulation (e.g., by including 3D sound). A final limitation might be the lack of body representation in the virtual environment. Body representation seems to enhance presence in virtual reality (Sanchez-Vives & Slater, 2005) but because of technical limitations, participants could not see their own body within the currently used spherical videos. Although none of the participants articulated irritations regarding the missing body, it seems interesting but also ambitious for future research to include a rendering of the own body into the virtual environment. While such procedure might enhance a feeling of presence, it also certainly requires an additional experimental phase to familiarize participants with this new situation.

Besides these limitations and the differences between virtual and real environments that were observed in the current study, we see great potential in the use of spherical videos as stimuli for social cognition research. Compared to 3D virtual reality environments, spherical videos are comparatively cheap and easy to generate. These videos can be presented using HMDs to allow for natural head and body movements and permit the acquisition of eye-tracking data that is not deteriorated by quickly changing light or weather conditions that can be encountered in real-life environments involving mobile eye-tracking (Niehorster, Cornelissen, Holmqvist, Hooge, & Hessels, 2017). Since our results indicate that the possibility for social interaction seems important for modulating social attention, it might be an interesting approach for future research to script spherical videos in order to effectively simulate such interaction. Although such approach seems demanding since the observer’s behavior is difficult to predict and would therefore require a precisely orchestrated scene, some basic aspects of social attention might well be simulated with such scripted videos. For example, a crucial aspect of social interaction is eye contact (Ellsworth et al., 1972; Wirth, Sacco, Hugenberg, & Williams, 2010), which could be simulated by purposefully looking into the camera at defined time points during the recording of the spherical video. Furthermore, it has been shown that social status is relevant for gaze allocation (Foulsham et al., 2010) but in this study, participants watched a group discussion on a desktop monitor “as if they were in the room”. Spherical videos could further enhance the external validity of such study designs. As another example to test the influence of norms, one can think of a setup similar to Risko and Kingstone (2011). They concealed the fact that they recorded eye movements by apparently switching off the eye tracker. This manipulation resulted in a substantial change in viewing behavior, presumably caused by a shift in social norms. Similarly, Cañigueral, Hamilton, and Ward (2018) also showed that wearing an eye tracker itself alters viewing behavior. Assuming compliance with ethical considerations, an HMD setup holds the opportunity to completely conceal eye-tracking. It is easy to implement with an HMD since the built-in eye tracker is usually not recognizable by laypersons. All in all, we feel that we have only touched the surface of what is possible with the usage of spherical videos for social cognition research. At the same time, several limitations of (interactive) eye tracking with unrestrained head movements are addressed (cf. Valtakari et al., 2021). We believe that this technique offers great potential for many research questions, especially since accessibility increases with the availability of spherical cameras and HMDs with included eye-tracking devices.

To sum up, this study examined the reliability and validity of spherical videos for examining social attention and it provided evidence for a reduction of gaze on other persons in real life as compared to laboratory conditions even when closely matching both environments. Viewing behavior was largely unaffected by social anxiety and autism spectrum traits but was modulated by the number of persons in the scene, especially when viewing spherical videos. In addition to these findings, we also observed general differences between virtual and real environments with respect to the stability of viewing patterns across locations and the spatial distribution of gaze proportions within the field of view. Despite these discrepancies, we believe that the use of HMDs and especially spherical videos holds great promise for social cognition research since they allow for a multimodal, contextually embedded, and dynamic stimulus presentation (Parsons et al., 2017; Risko et al., 2016; Zaki & Ochsner, 2009). However, the simulation of potential or actual social interactions in controlled laboratory research remains a challenging problem where, as discussed, spherical videos are only of limited help.

## Electronic supplementary material

Below is the link to the electronic supplementary material.

(PDF 258 KB)

## Footnotes

1 Spherical videos, also referred to as 360^∘^ videos, are videos recorded with multiple cameras to cover the whole surrounding at a specific location (see Methods section for more details). When we refer to spherical videos in the current article, we imply its presentation through an HMD with head tracking enabled. Please note that such spherical videos can also be watched on standard monitors and smartphones. Depending on the software, navigation is then enabled through keyboard, mouse, or device movements.

2 See the https://aspredicted.org/p7a83.pdfpreregistration for details.

3 Examples of the spherical videos from the different locations can be watched at https://www.youtube.com/playlist?list=PLFO679j3PTWpcFPRZ4i75_usOH7UwrQ4bhttps://www.youtube.com/playlist?list=PLFO679j3PTW https://www.youtube.com/playlist?list=PLFO679j3PTWpcFPRZ4i75_usOH7UwrQ4bpcFPRZ4i75_usOH7UwrQ4b.

4 Incorporating the environment and the location that participants started with into the analyses did not reveal statistically significant effects of these factors. Thus, order effects do not seem to constitute a source of error and were therefore neglected in the final set of analyses.

5 Since the residuals of the linear mixed model were not normally distributed, we ensured the validity of the present analysis by additionally calculating a robust linear mixed model (Koller, 2016). This robust model provided almost identical parameter estimates (see Table S1 in the supplementary material) and thus supports our interpretations.

6 See supplementary material for further details.
