# Supplementary material for: Reality in a sphere: A direct comparison of social attention in the laboratory and the real world
Source: Behav Res Methods. 2021 Dec 16;54(5):2286–301. doi: 10.3758/s13428-021-01724-0 (PMC9579106; doi:10.3758/s13428-021-01724-0)
Supplement: Supplementary file 1 — (PDF 258 KB) [file 13428_2021_1724_MOESM1_ESM.pdf]

Reality in a sphere: A direct comparison of social attention in the laboratory and the real world

Supplementary Material

Jonas D. Großekathöfer, Christian Seis, Matthias Gamer

1 Assumption check & robust LMM

A central assumption for the preregistered linear mixed models was not met by our data: the residuals were not normally distributed (see Figure S1, $W = 0.94$, $p < .001$).

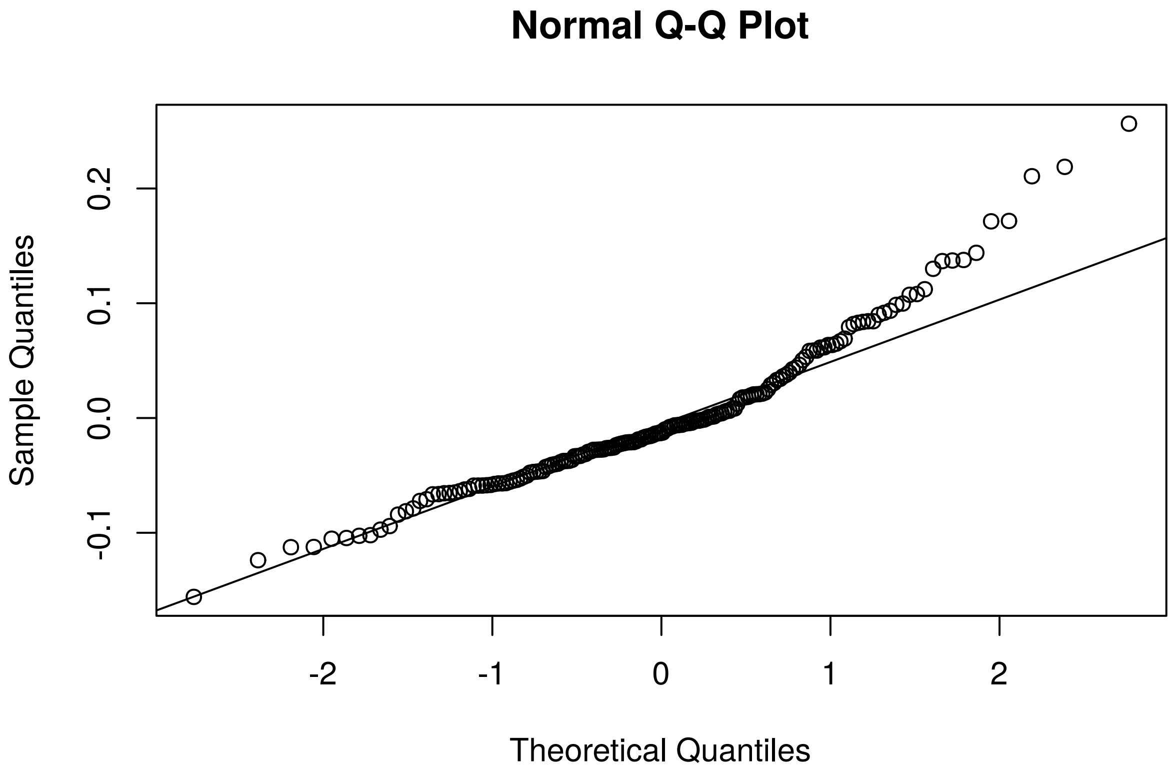

Figure S1: QQ plot for the residuals of the originally preregistered model.

Since this can have severe consequences for the statistical properties of the linear mixed model (Kenny and Judd 1986), we additionally calculated a robust linear mixed model following Koller (2016). These methods aim to provide robust estimates in conditions where assumptions for linear mixed models are violated. For the

Table S1: Estimated coefficients for a robust linear mixed model (Koller, 2016), specified exactly like the preregistered model 1 (see main article) with environment and ROI as fixed and participant ID as random effects for the prediction of gaze proportions.

	Estimate	*SE*	*t*
Intercept	0.19	0.00	38.03
Environment (RE)	-0.09	0.00	-18.63
ROI (object)	0.02	0.00	3.64
Environment (RE) × ROI (object)	0.02	0.00	3.09

Note. The robust linear mixed model is based on sum-to-zero contrasts. RE: real environment, ROI: region of interest.

Table S2: Estimated standard deviations, and correlations between the random-effects.

Groups	Name	SD	*r*
Subject (sub)	Intercept	0.03	
Location (loc)	Intercept	0.02	
	Present persons (P)	0.01	-1
Residual		0.13	

current study, the estimated coefficients of the robust model (see Table S1) closely matched the coefficients of the original linear mixed model (see Table 1 in the main article). As a result, the estimates, standard errors, and t-values are very similar and support our interpretation of the original findings in the main article.

2 Convergence issues in Model 4

Following the outlined model building path in the manuscript the complete model was initially specified[1] as:

$$fix \sim env * roi * P + (1|sub) + (1 + P|loc)$$

The correlation between location and present persons was estimated to be exactly -1 (see Table S2). Thus, the estimated correlation can be considered compromised, resulting in meaningless model output. In a subsequent step, we, therefore, suppressed the correlation between the random slope for present persons and locations, and specified the following pruned model (note the `||` indicating uncorrelated effects in the random term for `loc`):

$$fix \sim env * roi * P + (1|sub) + (1 + P||loc)$$

The model converged successfully and is reported in the manuscript (see Table 5).

[1] Using the `lme4` notation (Bates et al. 2015).

3 References

Bates, Douglas, Martin Mächler, Ben Bolker, and Steve Walker. 2015. "Fitting Linear Mixed-Effects Models Using lme4." *Journal of Statistical Software* 67 (1): 1–48. https://doi.org/10.18637/jss.v067.i01.

Kenny, David A., and Charles M. Judd. 1986. "Consequences of Violating the Independence Assumption in Analysis of Variance." *Psychological Bulletin* 99 (3): 422–31. https://doi.org/10.1037/0033-2909.99.3.422.

Koller, Manuel. 2016. "robustlmm: An R Package for Robust Estimation of Linear Mixed-Effects Models." *Journal of Statistical Software* 75 (6): 1–24. https://doi.org/10.18637/jss.v075.i06.
